# Impact of Preoperative Sinus Rhythm on Concomitant Surgical Ablation’s One-Year Success in Patients with Atrial Fibrillation: A Prospective Registry Cohort Study

**DOI:** 10.3390/jcm13195824

**Published:** 2024-09-29

**Authors:** Maximilian Vondran, Tamer Ghazy, Yeong-Hoon Choi, Taoufik Ouarrak, Bernd Niemann, Etem Caliskan, Nicolas Doll, Jochen Senges, Thorsten Hanke, Ardawan J. Rastan, CASE-AF Investigators

**Affiliations:** 1 Department of Cardiac and Vascular Surgery, Klinikum Karlsburg, Heart and Diabetes Center Mecklenburg-Western Pomerania, 17495 Karlsburg, Germany; 2 Department of Cardiac and Thoracic Vascular Surgery, Philipps-University Hospital, 35043 Marburg, Germany; tamer.ghazy@uk-gm.de (T.G.); a.rastan@uk-gm.de (A.J.R.); 3 Department of Cardiac and Vascular Surgery, Herz-Kreislauf-Zentrum, 36199 Rotenburg an der Fulda, Germany; 4 Department of Cardiovascular Surgery, Kerckhoff Klinik, 61231 Bad Nauheim, Germany; y.choi@kerckhoff-klinik.de; 5 Stiftung Institut für Herzinfarktforschung, 67063 Ludwigshafen, Germany; ouarrak@stiftung-ihf.de (T.O.); senges@stiftung-ihf.de (J.S.); 6 Department of Cardiovascular Surgery, University Hospital Giessen, 35392 Giessen, Germany; bernd.niemann@chiru.med.uni-giessen.de; 7 Department of Cardiovascular Surgery, Charité Universitätsmedizin Berlin, 13353 Berlin, Germany; ibrahim-etem.caliskan@dhzc-charite.de; 8 Department of Cardiac Surgery, Schuechtermann-Klinik, 49214 Bad Rothenfelde, Germany; ndoll@schuechtermann-klinik.de; 9 Department of Cardiac Surgery, Sana Heart Center, 70174 Stuttgart, Germany; 10 Department for Cardiac Surgery, Asklepios Klinikum Harburg, 21075 Hamburg, Germany; t.hanke@asklepios.com

**Keywords:** atrial fibrillation, cardiac surgery, surgical ablation, sinus rhythm, maze procedure

## Abstract

**Background**: The surgical ablation (SA) of atrial fibrillation (AF) during cardiac surgery is performed in only 8–40% of patients. We performed a subgroup analysis of the 1-year follow-up from the German CArdioSurgEry Atrial Fibrillation (CASE-AF) registry to determine how preoperative sinus rhythm (SR) prior to SA affected the outcomes. **Methods**: The CASE-AF registry enrolled AF patients scheduled for cardiac surgery with concomitant SA. The in-hospital and one-year follow-up data were collected prospectively and analyzed retrospectively. **Results**: From September 2016 to August 2020, 964 patients were enrolled in the CASE-AF registry. Among them, 333 patients were in SR immediately before surgery (study cohort). A complete follow-up was achieved for 95.6%. Both the severity of the AF (modified European Heart Rhythm Association symptom classification, *p* < 0.001) and the frequency of AF symptoms (*p* = 0.006) were significantly reduced at one year compared to the preoperative baseline. Almost 90 percent of the patients underwent left atrial appendage occlusion (LAAO) during the procedure. The one-year mortality (4.1%) and stroke rates (3.2%) were low. SR was evident in 70.3% of the patients at the one-year follow-up. **Conclusions**: Patients with AF who have SR at the time of surgery should not be excluded from SA, as it appears to be a safe and effective procedure.

## 1. Introduction

Approximately 6–30% of cardiac surgery patients present with concomitant atrial fibrillation (AF), depending on the underlying disease [1,2,3]. While undergoing cardiac surgery, only 8–48% of AF patients currently receive concomitant surgical ablation therapy (SA) [1,2,3]. However, the data on reduced all-cause mortality, long-term stroke rates, and improved quality of life for treated patients are superior to those for untreated AF patients [2,4,5,6,7]. Moreover, solid guideline recommendations exist [1,8,9]. However, many cardiac surgeons believe that AF patients do not benefit from additional SA when admitted or operated on in sinus rhythm (SR). In addition, some surgeons are concerned about the risk of concurrent SA, especially in patients who enter surgery with short-latency paroxysmal AF and a low disease burden. However, the structural abnormalities and remodeling processes associated with AF persist despite the transient SR, and deteriorate as the AF worsens [8].

There is a lack of data regarding the safety of concomitant SA in patients with AF who have SR prior to cardiac surgery. In a subgroup analysis of the nationwide, prospective, observational, multicenter German CArdioSurgEry Atrial Fibrillation Registry (CASE-AF), we already demonstrated the low perioperative risk profile of these patients [10]. However, conclusions about SA’s effectiveness were not feasible because of the short perioperative observational period.

Our subgroup analysis now reports on SA’s efficacy and the major adverse events during the one-year follow-up monitoring.

## 2. Patients and Methods

Full descriptions of the patients and methods, and detailed information on the CASE-AF registry and the follow-up modalities can be found in the main publications [11,12]. The main paper and a subgroup analysis by Grubitzsch and colleagues also provide detailed information on the operational techniques and line concepts used [11,13].

Conducted under the auspices of the Institute for Heart Attack Research (Institut für Herzinfarktforschung (IHF), Ludwigshafen, Germany), the CASE-AF registry is an enrollment-driven, prospective, nationwide, observational, multicenter study designed to collect data on the clinical outcomes of patients undergoing SA for AF according to their preoperative heart rhythm at admission [11]. The registry includes patients with AF and an underlying cardiac disease who are scheduled for surgery with concomitant SA or stand-alone SA. The central database maintained by the IHF allows for data to be uploaded via an electronic case report form (eCRF). Pre-, intra-, and postoperative data and the subsequent one-year outcomes are collected.

To conduct our subgroup analysis, we divided the entire CASE-AF registry cohort based on the patients’ heart rhythms when they entered the operating room. Our study included patients who were in SR before undergoing cardiac surgery with concomitant SA. The patients who did not enter the operating theatre in SR, had missing or inconclusive data, or underwent stand-alone SA were excluded from this study.

## 3. Follow-Up

AF relapse, arrhythmia documentation, hospital readmission, the modified European Heart Rhythm Association symptom classification (mEHRA) (Reflects AF-related symptoms and the patient’s perception of their general health. The mEHRA symptom score is defined as I: none; IIa: mild, normal daily activity not affected, symptoms not troublesome to patient; IIb: moderate, normal daily activity not affected but patient troubled by symptoms; III: severe, normal daily activity affected; and IV: disabling, normal daily activity discontinued) [14], the CHA2DS2-VASc score, electric or chemical cardioversion, redo ablation, cardiac implantable electronic device implantation, postoperative out-of-hospital complications, rehospitalization, current anticoagulation and/or antiarrhythmic drugs, and mortality data were collected at, but not limited to, the one-year follow-up. The IHF was responsible for central data monitoring, and initial queries detected in the eCRFs prevented further data entry until they were corrected. After data entry, dedicated IHF statisticians performed further analyses to detect inaccuracies and maintain data quality. The follow-up exams after one year were performed by a local center practice or by IHF staff. The protocol included telephone contact one year after discharge.

## 4. Study Endpoints

The primary study endpoint was cardiac rhythm one year after surgery. The secondary endpoints were major adverse events, no AF during follow-up (a combined endpoint of no AF recurrences at three months, no current AF, no re-ablation, no cardioversion, and no rehospitalization for AF), AF recurrence, and the all-cause mortality during the one-year follow-up. We also examined other adverse and heart-rhythm-specific outcome parameters during the one-year follow-up.

## 5. Statistics

The categorical variables were reported as numbers and percentages. A chi-squared or Fisher’s exact test was used for comparison, if necessary. The continuous variables were shown as the median and quartiles or the mean and standard deviation. All the tests were two tailed, and *p*-values < 0.05 were considered statistically significant. The SAS statistical software package version 9.4 (Cary, NC, USA) was used for all the analyses.

## 6. Ethical Considerations

The CASE-AF registry was registered in the ClinicalTrials.gov database (NCT03091452), and was approved by an Ethics Committee (Landesärztekammer Rheinland-Pfalz, ID: 837.536.15 [10304]). This study’s design, pseudonymized data collection, and data publication adhered to the tenets of the Declaration of Helsinki. Written informed consent was obtained from each included patient.

## 7. Results

Eighteen German cardiac surgery centers enrolled 964 consecutive patients between September 2016 and August 2020. First, we excluded 110 patients presenting with stand-alone SA, and 521 patients due to their having AF as their heart rhythm when entering the operating room. Finally, 333 patients were in SR immediately before surgery (study cohort). The time from the procedure to follow-up was 449 (390, 575) days. We managed complete follow-ups in 95.5% of the patients.

Table 1 summarizes the preoperative patient characteristics. Approximately half of the patients were at least moderately or more severely limited by AF. The type of AF was paroxysmal in 79.0%, persistent in 15.3%, and long-persistent in 5.7%, respectively. In addition, 9.0% of the patients had a previous unsuccessful ablation procedure, and 13.1% had AF refractory to amiodarone. Moreover, the main underlying cardiac disease was valvular, and the mean LA diameter was 47 ± 9 mm.

Table 2 illustrates the periprocedural details. Almost 90 percent of all the patients underwent a left atrial appendage occlusion (LAAO) during the operation. Our cohort underwent more epicardial and radiofrequency ablations. The surgeons took a minimally invasive approach in 21.0% of the patients. Almost 60% of the patients underwent a box lesion, with just under half receiving additional left arterial lines and approximately 10% RA lines. Immediately after the procedure, 92.0% of the patients experienced SR.

Table 3 illustrates the heart rhythms documented after one year of follow-up and the occurrence, timing, and clinical manifestation of AF recurrences. Twelve months after cardiac surgery plus SA, 70% of the patients were in SR. More than 70% of the patients presented with no symptoms, and 27% said their daily activities were only mildly affected. Among those who had symptoms, they were less frequent than before surgery. However, 65.6% of the patients had no AF recurrence (a combined endpoint of no AF recurrences at three months, no current AF, no re-ablation, no cardioversion, and no rehospitalization for AF).

Table 4 shows the rate of adverse events during the 12-month follow-up period after surgery with additional SA. It also includes the one-year mortality and reveals a very low incidence of complications.

**Figure 1:**
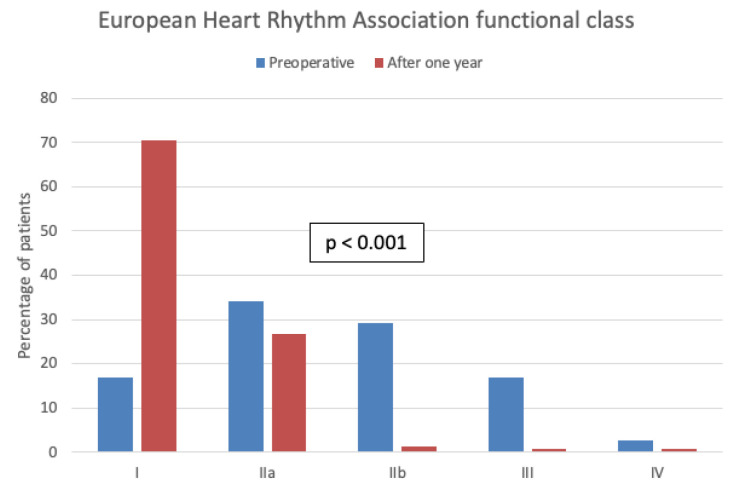
Modified European Heart Rhythm Association functional class preoperative and after one year of follow-up.

**Figure 2:**
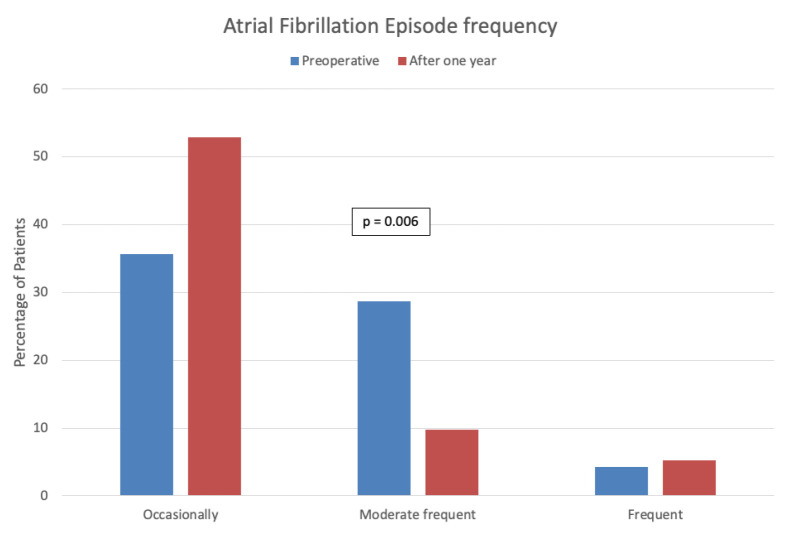
Atrial fibrillation episode frequency preoperative and after one year of follow-up.

In addition, there was a significant decrease in symptom intensity (Figure 1) and frequency (Figure 2) after one year of follow-up compared to preoperatively.

Table 5 shows the rehospitalization rates and the reasons for rehospitalization. It also illustrates the specific procedures performed. Approximately 15% of the rehospitalizations were AF related. However, the proportion of repeat cardioversions, the rate of reoperations, and the rate of repeat ablations were low.

Table 6 presents the 12-month follow-up data only for patients with concomitant SA and mitral ± tricuspid valve surgery or aortic valve surgery or coronary artery bypass graft surgery. The one-year all-cause mortality rates were 3.2%, 1.6%, and 5.6%, respectively. In addition, 1.4%, 2.0%, and 6.0% of patients were at least moderately affected by their AF in their daily lives.

## 8. Discussion

In summary, in our study, cardiac surgery with additional SA in patients with AF who were in SR before surgery appears to be a safe and effective procedure, even after one year of follow-up. In addition, it leads to a significant reduction in symptom intensity and frequency compared to preoperatively, with low AF recurrence rates.

The German CASE-AF registry, as a purely surgical AF ablation registry, is the first to enable insight into the reality of everyday practice concerning concomitant SA in Germany as a real-life, all-comers registry beyond the ideal of randomized controlled trials. Of the 78 existing German centers, 18 participated in our CASE-AF registry. Thus, almost a quarter of all German centers participated in this registry. In Germany, about 93,000 cardiac surgical procedures, in the classical sense (catheter interventional valve procedures excluded), are performed annually [15]. The proportion of patients who receive an SA during their surgery is 4.4% [15]. Assuming that approximately 20% of cardiac surgery patients suffer from AF [1,2,3], only a minority of AF patients receive an SA. Between 2016 and 2020, about 1000 concomitant SAs were documented by the participating CASE-AF centers. During our observation period, at least 15,000 patients could have presented with a potential indication for additional SA during cardiac surgery in Germany. Even if we speculate that there are undocumented cases, our registry mirrors the “real world” situation of cardiosurgical practice, which, unfortunately, still reveals a certain reluctance to perform ablations in Germany.

Paroxysmal AF patients who experience AF-related symptoms briefly experience those symptoms and limitations in everyday life significantly more intensely and stressfully than patients with persistent AF [16]. Surprisingly, in our work, almost 50 percent of the patients who were in sinus rhythm before surgery were limited by their atrial fibrillation, ranging from moderate to disabling.

We found it impressive that, in addition to our good rhythm outcome, 71% were no longer suffering AF-related limitations (mEHRA class I) after one year of follow-up. An additional 26% of the patients were only slightly limited (mEHRA class IIa) daily. Thus, after undergoing surgery with SA, only about 3% had an mEHRA class ≥ IIb, compared with about 50% of the patients preoperatively (Figure 1). We can assume that ablation also resulted in a considerable increase in quality of life, even though we employed no tool to assess the quality of life in our registry.

**Figure 3:**
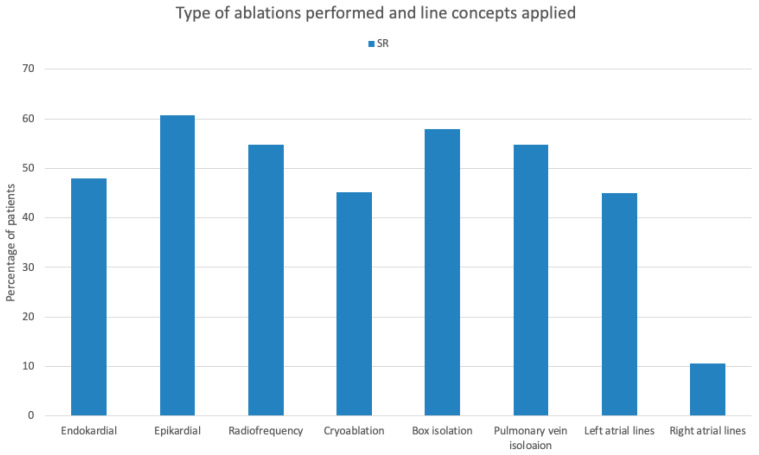
Type of ablation performed and line concepts applied.

Moreover, our work has given us a better impression of the reality of the applied lesion sets in the context of concomitant SA in Germany. Indeed, our patient group was very heterogeneous, but it still allowed for in-depth reflection and showed the potential for improvement in daily practice. Our patients were characterized by paroxysmal AF and a high proportion of coronary artery disease as the underlying cardiac pathology. The rate of mitral valve surgery was low (36.7%). Looking at the lesion sets they underwent, they tended to undergo a pulmonary vein isolation (PVI) or box lesion; 45% received additional LA lines and 10% additional RA lines (Figure 3). Furthermore, almost 90% of our patients received a left atrial appendage occlusion (LAAO).

The ablation concepts documented by our study can be considered appropriate and justifiable considering that for the high proportion of patients with paroxysmal AF and a few mitral valve patients, most of the operations were probably performed without opening the atria. The PRAGUE-12 multicenter randomized controlled trial (RCT) patient population was similar to those of the CASE-AF registry and our subgroup [17]. Moreover, patients undergoing coronary artery bypass grafting (CABG) and valve surgery were included in that RCT. However, that work had no uniform lesion set or ablation energy concept, so the SR rate at the one-year follow-up was only 60.2% in the AF patients with SA [17]. In addition, in an RCT on CABG patients with non-paroxysmal AF and concomitant SA, the SR rate at one year was 83%. The patients to be ablated were additionally randomized into a group with a mini-maze and a group with PVI. However, there was no difference in the SR between the different line concepts after one year [18]. Nevertheless, the bi-atrial Cox maze III/IV procedure in these patients would have resulted in an over 90% probability of SR at five years [19]. Also, PVI with LAAO alone can result in SR in 50–80% of those in similar patient groups [1,20,21]. Our one-year results, with over 70% of the patients in SR, are similar to the expected outcome. With the proportion of minimally invasive surgery in our patients at 21%, this may also be why relatively few LA, and even fewer RA, lines have been added to the box lesion.

In contrast, in patients with non-paroxysmal AF, a bi-atrial maze lesion concept is recommended for all operations [1,22]. More than two-thirds of these patients would still be in SR seven years later, despite presenting with a more complex AF pathology [23]. Only patients with non-paroxysmal AF undergoing mitral valve surgery were described in an RCT by Gilinov and colleagues. In addition, their ablated patients were randomized again into a group with a bi-atrial maze procedure and one with PVI only. Their SR rate was 63.2% at the one-year follow-up. However, the SR rates did not differ between the line concepts after one year of follow-up [4]. Indeed, not every patient needs a bi-atrial maze procedure despite its being the gold standard [1,22]; nevertheless, at least a complete LA maze procedure is advisable [24]. In the future, more attention should be paid to the selection of the lesion set in order to achieve the best possible results. In addition, in patients with concomitant SA, closer rhythm monitoring would be desirable, e.g., for the early detection and treatment of possible early recurrences. To this end, the perioperative implantation of an implantable loop recorder could be considered on an individual patient basis, and the increasingly sophisticated non-invasive rhythm recording using smartwatches could be utilized more in the diagnosis and monitoring of these patients.

The possibility of a new permanent pacemaker implantation (PPI) discourages concomitant SA in subjectively ineligible AF patients. In our work, we observed a new PPI rate of approximately 11% in both groups one year after cardiac surgery plus SA. This figure is put into perspective by comparing our “real-world” registry data with data from a recent meta-analysis [25], which included only RCTs. In that study, the PPI rates at one year varied from 0 to 21.5%, with the included studies revealing the broad heterogeneity of the operations performed, the lesion sets, the underlying cardiac disease, and the duration of AF [25]. Gilinov and colleagues reported the highest PPI rate in that meta-analysis. The PPI rate in their study was also about 10% after one year in the group of patients without SA [4]. In the work of Budera et al., a study of CABG and valvular patients, surprisingly, patients without SA showed a higher new PPI rate at one year (13% vs. 9.9%) [17].

However, permanent right ventricular pacing is suspected of triggering a higher incidence of AF, heart failure, and mortality [26,27,28]. Nevertheless, two studies in which the perioperative rate of new PPI ranged from 7.6 to 11.0% still demonstrated a long-term survival benefit among the ablated AF patients compared to the non-ablated group [2,7]. Patients with adequate sinoatrial node function usually do not need a new PPI after SA, but require more time for SR recovery [29]. However, these patients unnecessarily receive a new PPI to shorten their hospital stay. SA probably plays a minor role in the subsequent need for a new PPI; the surgical technique and surgeon’s experience appear to play a much more significant role [30,31]. However, we can only speculate about the reasons for our new PPIs, and whether the RA lines triggered sinus node dysfunction or higher-grade atrioventricular blocks were caused by a mixture of factors, including elderly patients, multiple valve surgery, and valve replacements. Using the CASE-AF registry patient population, the factors predispose individuals to require a new PPI after SA are being analyzed in an additional subgroup analysis.

## 9. Limitations

The fact that only patients with AF were enrolled in the CASE-AF registry is the main limitation of this study. With our registry data, it is impossible to establish a control group for comparison with non-ablated patients in SR. Moreover, the serial and Holter electrocardiograms we relied on to detect long-term arrhythmia might not always provide accurate rhythm data after ablative procedures, compromising our ability to interpret the results at the one-year follow-up adequately. This factor could lead to misinterpreting success. In addition, the ablative procedures were carried out according to the local centers’ routine practices or surgeon’s preference. Although patients were continuously and prospectively enrolled in this study and the data were validated by an independent body (IHF), the registry’s design does not preclude sample bias. Most of this study’s other limitations are inherent to its observational design.

## 10. Conclusions

A concomitant SA demonstrated safety and entailed a low risk of perioperative and one-year mortality and morbidity. A simultaneous SA during cardiac surgery in AF patients entering the operating room in SR appeared to yield a relatively high success rate during a one-year follow-up, coupled with a modest AF recurrence rate. Preoperative SR in AF patients may not necessarily serve as a strong counterargument against considering concomitant SA in AF patients.

## Figures and Tables

**Table 1:** Preoperative patient characteristics.

Variable	Preoperative SR (*n* = 333)	
Age, y	67.3	±10.5
BMI, kg/m^2^	27.9	±4.8
Female	30.6	
Congestive heart failure	40.3	
Diabetes	20.4	
Hypertension	76.3	
Peripheral vascular disease	8.1	
Prior CVA	9.3	
Type of AF		
Paroxysmal	79.0	
Persistent	15.3	
Long persistent	5.7	
mEHRA classification		
I (No)	17.0	
IIa (Mild)	34.1	
IIb (Moderate)	29.2	
III (Severe)	17.0	
IV (Disabling)	2.7	
AF episode frequency		
Occasionally	35.7	
Moderately frequent	28.9	
Frequent	4.3	
Undecided	31.1	
Amiodarone refractory AF	13.1	
Any previous unsuccessful ablation therapy	9.0	
Left ventricular ejection fraction, %	55	±11
Left atrial diameter, mm	47	±9
CHA2DS2-Vasc score	3.1	±1.6
Underlying cardiac disease		
Coronary artery disease	38.3	
Valvular heart disease	56.9	
Other	5.4	

Categorical variables are presented as percentages, and continuous parameters as mean ± standard deviation. Abbreviations: AF, atrial fibrillation; CVA, cerebrovascular accident; mEHRA, modified European Heart Rhythm Association symptom classification; SR, sinus rhythm.

**Table 2:** Periprocedural details.

Variable	Preoperative SR (*n* = 333)	
Surgical access		
Median sternotomy	79.0	
Lateral thoracotomy	19.8	
Thoracoscopic	1.2	
Conversion to median sternotomy necessary	0.6	
Procedures performed		
CABG	45.2	
Mitral valve reconstruction	28.3	
Aortic valve replacement	27.7	
Tricuspid valve reconstruction	9.0	
Mitral valve replacement	8.4	
Aortic surgery	4.8	
Aortic valve reconstruction	1.2	
Tricuspid valve replacement	0.3	
Any atrial septal closure	3.9	
Energy delivered		
Endocardial	47.9	
Epicardial	60.8	
Energy source used for ablation		
Radiofrequency	54.8	
Maximum output, watt	37	±22
Total duration, sec	217	±184
Cryoablation	45.2	
Minimal temperature, °C	−69	±15
Total duration, sec	526	±272
Concept of ablation lines		
Box isolation	57.9	
Pulmonary vein isolation	54.7	
Left atrial lines	45.0	
Right atrial lines	10.6	
Left atrial appendage isolation	89.8	
Excision	37.1	
Endocardial suture	13.0	
Epicardial suture	15.4	
Epicardial ligature	2.0	
Other	0.3	
Stapler	22.7	
Exclusion via clip	24.1	
Atriclip	97.2	
Other	2.8	
Heart rhythm immediately after surgery		
Sinus rhythm	92.0	
Atrial tachycardia	1.4	
Atrioventricular block, 2nd degree	1.4	
Atrioventricular block, 3rd degree	5.1	

Categorical variables are presented as percentages, and continuous parameters as mean ± standard deviation. Abbreviations: CABG, coronary artery bypass grafting; SR, sinus rhythm.

**Table 3:** Heart rhythm, atrial fibrillation recurrences, symptom severity, and medical treatment during 12 months of follow-up.

Variable	Preoperative SR (*n* = 333)
Heart rhythm 12 months after surgery	
Sinus rhythm	70.3
Atrial fibrillation	29.7
Paroxysmal	16.1
Persistent	9.8
Long-term persistent	3.8
AF recurrence after three months and/or current AF	34.4
No AF during follow-up *	67.2
AF recurrence	
During the blanking period (≤3 months)	23.6
Symptomatic	15.1
Asymptomatic	11.2
After the blanking period (>3 months)	25.7
Symptomatic	16.4
Asymptomatic	13.3
Arrhythmia diagnostics	
Resting ECG	80.3
Long-term ECG (24 h)	74.2
Cardiac electrical device testing	3.7
mEHRA classification	
I (No)	70.5
IIa (Mild)	26.7
IIb (Moderate)	1.2
III (Severe)	0.8
IV (Disabling)	0.8
AF episode frequency	
Occasionally	52.9
Moderately frequent	9.8
Frequent	5.2
Undecided	32.0
Medication	
Antiarrhythmic drugs	
Class I	2.7
Class II	83.1
Class III	8.0
Digitalis	2.7
Anticoagulation	56.8
DOAC	65.3
VKA	33.5
Antiplatelet therapy	36.2

Categorical variables are presented as percentages. Abbreviations: AF, atrial fibrillation; DOAC, direct oral anticoagulants; ECG, electrocardiogram; mEHRA, modified European Heart Rhythm Association symptom classification; SR, sinus rhythm; VKA, vitamin K antagonist; *, combined endpoint of no AF recurrences at three months + no current AF + no re-ablation + no cardioversion + no rehospitalization for AF.

**Table 4:** Adverse events during 12-month follow-up.

Variable	SR (*n* = 318)
Post-hospital follow-up period *	
Cerebrovascular accident	2.0
Transient ischemic attack	0.3
Severe bleeding	0.3
Pericardial effusion	0.3
Phrenic nerve palsy	1.0
New pacemaker implantation w/o ICD or CRT	5.7
New pacemaker implantation	7.4
After 12 months of follow-up **	
Cerebrovascular accident	3.2
Transient ischemic attack	1.8
Severe bleeding	1.1
Pericardial effusion	3.9
New pacemaker implantation w/o ICD or CRT	10.9
New pacemaker implantation	12.9
All-cause one-year mortality **	4.1

Categorical variables are presented as percentages. Abbreviations: CRT, cardiac resynchronization therapy; ICD, implantable cardioverter–defibrillator; SR, sinus rhythm; *, in-hospital outcome not included; **, in-hospital outcome included.

**Table 5:** Rehospitalization during 12 months of follow-up.

Variable	SR (*n* = 318)	
Rehospitalization since discharge	34.3	
Reason for rehospitalization		
Cardiosurgical	6.9	
AF	14.7	
Other cardiovascular reason	15.7	
Non-cardiovascular reason	34.3	
Unknown	28.4	
Redo cardiac surgery	2.5	
Redo ablation	2.4	
Percutaneous	57.1	
Surgical	14.3	
Repeat cardioversion	4.8	
Number of cardioversions	1.0	(1.0, 1.5)

Categorical variables are presented as percentages and continuous parameters as median and quartiles. Abbreviations: AF, atrial fibrillation; SR, sinus rhythm.

**Table 6:** Twelve-month follow-up data only for patients with concomitant surgical ablation and mitral ± tricuspid valve surgery or aortic valve surgery or coronary artery bypass graft surgery.

Variable	MV ± TV Surgery (*n* = 94)	Isolated AV Surgery (*n* = 61)	Isolated CABG Surgery (*n* = 108)
All-cause one-year mortality **	3.2	1.6	5.6
Post-hospital follow-up period *			
Cerebrovascular accident	1.1	0.0	3.0
Transient ischemic attack	0.0	1.7	0.0
Severe bleeding	0.0	0.0	1.0
New pacemaker implantation w/o ICD or CRT	2.2	6.8	6.0
New pacemaker implantation w/ ICD or CRT	2.2	10.2	6.9
Rehospitalization since discharge	30	32.8	38.6
mEHRA class > IIb	1.4	2.0	6.0

Abbreviations: AV, aortic valve; CABG, coronary artery bypass grafting; CRT, cardiac resynchronization therapy; mEHRA, modified European Heart Rhythm Association symptom classification; ICD, implantable cardioverter–defibrillator; MV, mitral valve; TV, tricuspid valve; *, in-hospital outcome not included; **, in-hospital outcome included.

## Data Availability

Upon request to Ms. Belgin Özdemir, who can be reached at oezdemir(at)stiftung-ihf.de, the anonymized data used to support the results of this study may be released.

## Abbreviations

AF	Atrial Fibrillation.
CASE-AF Register	German CArdioSurgEry Atrial Fibrillation Register.
CABG	Coronary Artery Bypass Graft Surgery.
CI	Confidence Interval.
EuroSCORE	European System for Cardiac Operative Risk Evaluation.
IHF	Institute for Heart Attack Research (German: Institut Für Herzinfarktforschung).
LA	Left Atrial.
LAA	Left Atrial Appendage.
LAAOS III	The Left Atrial Appendage Occlusion Study III.
MACCE	Major Adverse Cardiac and Cerebrovascular Events.
mEHRA	Modified European Heart Rhythm Association symptom classification.
OAC	Oral Anticoagulation.
OR	Odds Ratio.
PPI	Permanent Pacemaker Implantation.
RA	Right atrial.
RCT	Randomized Controlled Trial.
SA	Surgical Ablation Therapy.
SR	Sinus Rhythm.
